# The HIF-1 Hypoxia-Inducible Factor Modulates Lifespan in *C. elegans*

**DOI:** 10.1371/journal.pone.0006348

**Published:** 2009-07-27

**Authors:** Yi Zhang, Zhiyong Shao, Zhiwei Zhai, Chuan Shen, Jo Anne Powell-Coffman

**Affiliations:** Department of Genetics, Development, and Cell Biology, Iowa State University, Ames, Iowa, United States of America; Massachusetts General Hospital/Harvard Medical School, United States of America

## Abstract

During normal development or during disease, animal cells experience hypoxic (low oxygen) conditions, and the hypoxia-inducible factor (HIF) transcription factors implement most of the critical changes in gene expression that enable animals to adapt to this stress. Here, we examine the roles of HIF-1 in post-mitotic aging. We examined the effects of HIF-1 over-expression and of *hif-1* loss-of-function mutations on longevity in *C. elegans*, a powerful genetic system in which adult somatic cells are post-mitotic. We constructed transgenic lines that expressed varying levels of HIF-1 protein and discovered a positive correlation between HIF-1 expression levels and lifespan. The data further showed that HIF-1 acted in parallel to the SKN-1/NRF and DAF-16/FOXO transcription factors to promote longevity. HIF-1 over-expression also conferred increased resistance to heat and oxidative stress. We isolated and characterized additional *hif-1* mutations, and we found that each of 3 loss-of-function mutations conferred increased longevity in normal lab culture conditions, but, unlike HIF-1 over-expression, a *hif-1* deletion mutation did not extend the lifespan of *daf-16* or *skn-1* mutants. We conclude that HIF-1 over-expression and *hif-1* loss-of-function mutations promote longevity by different pathways. These data establish HIF-1 as one of the key stress-responsive transcription factors that modulate longevity in *C. elegans* and advance our understanding of the regulatory networks that link oxygen homeostasis and aging.

## Introduction

During development, homeostasis, and disease, cells integrate diverse environmental inputs and implement the appropriate changes in gene expression to survive stresses or execute developmental programs. Of the many environmental challenges that animals encounter, oxygen deprivation (hypoxia) is of particular concern. Oxygen is the final electron acceptor in cellular respiration, and is, therefore, necessary for most metazoan life. Successful adaptation to hypoxia involves changes in genetic programs that modulate metabolism, cell death, growth, and cellular differentiation. Many of these processes also have roles in cellular aging. This suggests that the networks that govern oxygen homeostasis and aging may be intertwined, but the molecules and mechanisms that underpin these networks are not fully understood.

In animals as diverse as humans and *C. elegans*, the hypoxia-inducible factor (HIF) heterodimeric transcription factors are responsible for most of the hypoxia-induced changes in gene expression [1], [2]. HIFs have been studied intensively by the biomedical research community, because HIFs have central roles in cancer and cardiovascular disease. Small molecules that inhibit HIF may be effective chemotherapeutic agents. Conversely, for patients in which injury, anemia, or ischemia has decreased oxygen supply to tissues, treatments that increase HIF levels may improve cell survival and stimulate angiogenesis.

HIF regulatory networks are acutely sensitive to oxygen. The HIF transcription complex is heterodimeric, and both subunits belong to the family of DNA-binding transcription factors that contain basic-helix-loop-helix and PAS domains. HIFα stability is regulated by oxygen-dependent degradation. When oxygen levels are sufficiently high, HIFα is hydroxylated by members of the EGL-9/PHD family of Fe(II)- and 2-oxoglutarate-dependent dioxygenases. This modification allows VHL, a ubiquitin E3 ligase, to bind HIFα and target it for proteasomal degradation. In hypoxic conditions, HIFα is stable, and the HIF complex activates a battery of genes that enable adaptation to low oxygen conditions. HIF stability and activity are influenced by reactive oxygen species, citric acid cycle intermediates, Fe(II) availability, and intracellular signaling events [1], [3]. HIFs are, therefore, well-positioned to integrate information about oxygen availability and cellular redox and to effect changes that influence stress resistance.

Here, we test the hypothesis that HIF modulates post-mitotic aging. Previous studies in human cells had demonstrated that HIF promoted telomerase expression [4] and that this contributed to increases in the replicative lifespan of primary human lung fibroblasts in hypoxic conditions [5], but the roles of HIF in post-mitotic aging were not understood. The somatic cells of adult *C. elegans* are post-mitotic, and the rate of aging can be modulated by genes that control insulin-like receptor signaling, mitochondrial function, protein folding, or responses to reactive oxygen species and other stresses [6], [7]. The *C. elegans* genome encodes a single HIF alpha subunit, *hif-1*, and mutants lacking a functional *hif-1* gene are viable in normal culture conditions [8], [9]. In this study, we report that over-expression of HIF-1 protein extends the lifespan of *C. elegans* in a dose-dependent manner. Interestingly, we find that loss-of-function mutations in *hif-1* gene can also increase *C. elegans* longevity and stress tolerance. The HIF-1 over-expression and *hif-1* loss-of-function longevity phenotypes are not equivalent, as they differ in their dependence on *daf-16*/FOXO and *skn-1*/NRF2. These data provide insights to the intersection between oxygen homeostasis and aging, and they suggest that post-mitotic aging may be influenced by treatments or mutations that alter HIF-1 signaling.

## Results

### HIF-1 over-expression causes dose-dependent extension of adult lifespan

**Figure 1:**
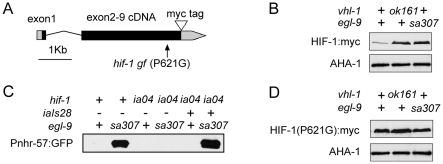
Characterization of the *hif-1* and *hif-1(P621G)* transgenes. (A) Diagram of the minigenes that express epitope-tagged HIF-1. Genomic sequence including the *hif-1* promoter sequence, exon 1, and intron 1, was fused to cDNA including exons 2–9 for the predominant *hif-1* mRNA isoform (*hif-1a*). A myc epitope tag was inserted. A proline to glycine conversion at amino acid residue 621 prevents VHL-1-mediated degradation [17]. (B) Representative protein blots show that epitope-tagged HIF-1 is stabilized by loss-of-function mutations in *vhl-1* or *egl-9*. AHA-1 is ortholgous to HIFβ/ARNT [48], and the abundance of AHA-1 does not vary significantly in these strains. (C) Western blots show the transgene restores HIF-1-mediated gene expression. *Pnhr-57*:GFP is a reporter for HIF-1 activity [20]. Its expression is dramatically reduced in *hif-1(ia04)* mutants and is restored by the *iaIs28* integrated *hif-1* transgene. (D) Oxygen-dependent degradation via the *vhl-1/egl-9* pathway is abolished when the HIF-1 proline residue 621 is mutated to glycine.

A small group of transcriptional regulators lying in the core of the stress response network, including DAF-16, SKN-1, SIR-2.1, PHA-4 and HSF-1, had been shown to extend lifespan when activated or over-expressed in transgenic *C. elegans* [10], [11], [12], [13], [14], [15], [16]. To determine whether HIF-1 had similar effects, we generated a series of transgenic animals with extra copies of *hif-1* integrated into the genome. The *hif-1* minigene is a fusion of genomic and cDNA sequences, and it includes a c-myc tag (Fig. 1A). We confirmed that the epitope-tagged HIF-1 protein, like endogenous HIF-1, was over-expressed in animals that lacked *vhl-1* or *egl-9* (Fig. 1B). Further, the transgene was able to restore expression of a HIF-1-dependent reporter, *Pnhr-57*:GFP, to a *hif-1* deletion mutant (Fig. 1C). In two independent lines (*iaIs27* and *iaIs28*), the *hif-1* integrated transgenes conferred moderate lifespan extension (22 or 23 days, *p*<0.0001, Tables 1 and S1).

**Table 1:** Over-expression of HIF-1 protein or *hif-1* loss-of-function mutations extend adult lifespan in *C. elegans* in standard lab culture conditions.

strain	life span mean±S.E.	Percentage *v*. N2	n	*p* value*	number of exp
N2 wild-type	20.0±0.3		166		4
*iaIs27 [Phif-1::hif-1::myc]*	22.3±0.4	12	109	<0.0001	3
*iaIs28 [Phif-1::hif-1::myc]*	23.0±0.3	15	131	<0.0001	3
*iaIs32 [Phif-1::hif-1(P621G)::myc]*	25.9±0.6	30	117	<0.0001	3
*iaIs33 [Phif-1::hif-1(P621G)::myc]*	24.1±0.5	20	125	<0.0001	3
*iaIs34 [Phif-1::hif-1(P621G)::myc]*	26.7±0.4	33	125	<0.0001	3
*hif-1(ia04)*	24.1±0.5	20	147	<0.0001	4
*hif-1(ia07)*	27.0±0.6	35	56	<0.0001	2
*hif-1(ok2564)*	22.4±0.6	12	74	<0.0001	2
*egl-9(sa307)*	20.8±0.3	4	126	0.2	4
*egl-9(sa307);hif-1(ia04)*	19.2±0.3	−4	134	0.3	3

* The longevity of each strain was compared to wild-type, and the *p* values were calculated by log-rank tests.

**Figure 2:**
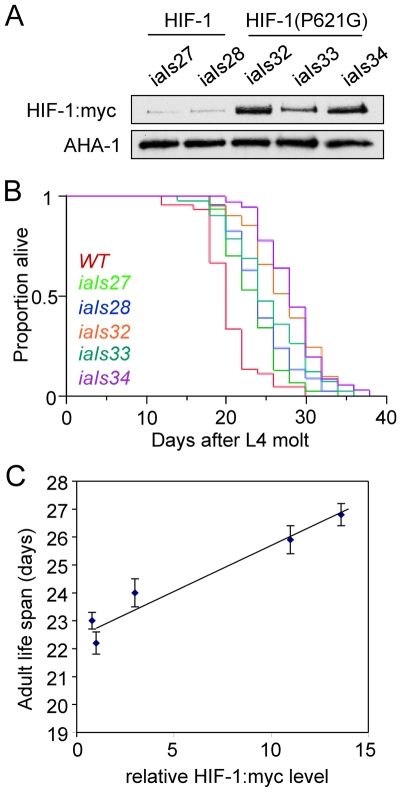
HIF-1 over-expression extends longevity in a dose-dependent manner. (A) Protein blots quantitate expression of HIF-1 and HIF-1(P621G) transgenes (tagged with the myc epitope). *iaIs27* and *iaIs28* contain integrated copies of the *hif-1* minigene. *iaIs32*, *iaIs33*, and *iaIs34* carry integrated copies of *hif-1 (P621G)*. (B) Strains carrying *hif-1* transgenes live longer (*p*<0.0001). Proportion alive is plotted over time. (C) The mean adult lifespans of strains over-expressing HIF-1 are positively correlated with the HIF-1 expression levels. Error bars represent the standard errors of the means.

To further test the hypothesis that HIF-1 over-expression increased lifespan in *C. elegans*, we constructed a transgene in which HIF-1 proline 621 was converted to glycine. In wild-type animals, HIF-1 proline 621 is hydroxylated by EGL-9, and this covalent modification had been shown to mediate binding of HIF-1 to VHL-1 [17]. Mutations in *vhl-1* or *egl-9* can also be used to inhibit HIF-1 degradation, but these mutations have other defects that can complicate interpretation of the phenotypes. The *vhl-1(ok161)* deletion causes some changes in extracellular matrices that are not suppressed by a mutation in *hif-1* [18], and animals carrying strong loss-of-function mutations in *egl-9* have an array of morphological and behavioral defects [19], [20], [21], [22]. To understand the effects of HIF-1 over-expression, we generated transgenic animals carrying the *hif-1 (P621G)* expression construct and analyzed 3 independently isolated lines (*iaIs32, iaIs33*, and *iaIs34*). As shown in Fig. 1D, the HIF-1(P621G) gain-of-function mutation released the protein from degradation via the *egl-9/vhl-1* pathway. In these strains, the HIF-1 (P621G) protein was expressed at levels 3–14 times greater than HIF-1 expressed from transgenes without the stabilizing mutation (Fig. 2A). The *hif-1 (P621G)* lines lived 20%–34% longer than wild-type N2 worms (Fig. 2B, Table 1). Moreover, the mean adult lifespans of the five *hif-1* transgenic strains were proportionately correlated with HIF-1 expression levels (Fig. 2C). These data established that HIF-1 over-expression caused dose-dependant lifespan extension.

### HIF-1 over-expression acts in parallel to DAF-16 and SKN-1 to extend lifespan

**Figure 3:**
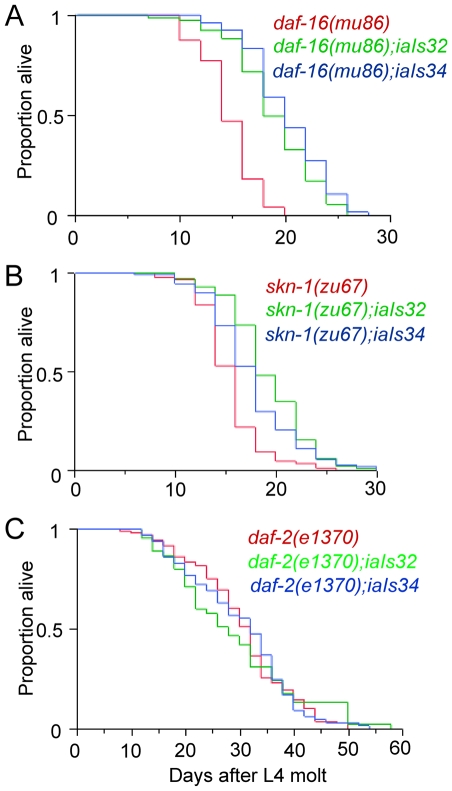
HIF-1 over-expression extends the lifespan of *daf-16*-deficient and *skn-1*-deficient mutants. (A, B) *hif-1(P621G)* transgenes (*iaIs32* or *iaIs34*) extend the lifespan of animals carrying loss-of-function mutations in (A) *daf-16* (*p*<0.0001) or (B) *skn-1* (*p*<0.0001). (C) The longevity of *daf-2(e1370)* animals carrying a *hif-1 (P621G)* transgene is equivalent to that of *daf-2(e1370)* single mutants.

Mutations in the *daf-2* insulin-like receptor gene increase nuclear localization of the DAF-16 FOXO transcription factor and dramatically increase longevity and resistance to heat or oxidative stress. Conversely, *daf-16* loss-of-function mutants age quickly [10], [13], [23], [24], [25], [26]. We considered the possibility that loss-of-function mutations in *daf-16* might be epistatic to HIF-1 over-expression in longevity assays. As shown in Fig. 3A and Table 2, *hif-1(P621G)* transgenes increased the mean lifespan of *daf-16 (mu86)* mutants by up to 33%. This was very similar to the effect that HIF-1(P621G) had on wild-type animals (Table 1). These data show that in animals over-expressing HIF-1, DAF-16 and HIF-1 act in parallel to influence post-mitotic aging.

**Table 2:** Effects of HIF-1 over-expression or *hif-1* loss-of-function mutations on the longevity of *daf-16* or *skn-1* mutants.

genotype	Adult life span mean±S.E.	Percentage *v*. control	n	*p* value	number of exp
*daf-16(mu86)*	15.1±0.2		191		5
*daf-16(mu86); hif-1(ia04)*	14.8±0.3	−2	105	0.3*	3
*daf-16(mu86); iaIs32*	19.0±0.4	27	77	<0.0001*	2
*daf-16(mu86); iaIs34*	20.2±0.4	33	78	<0.0001*	2
*skn-1(zu67)*	15.4±0.3		87		2
*skn-1(zu67);hif-1(ia04)*	14.8±0.4	−4	76	0.5†	2
*skn-1(zu67); iaIs32*	19.2±0.4	25	98	<0.0001†	2
*skn-1(zu67); iaIs34*	17.6±0.4	14	108	<0.0001†	2
*daf-2(e1370)*	32.5±1.1		76		3
*daf-2(e1370); hif-1(ia04)*	34.0±1.2	5	75	0.2‡	3
*daf-2(e1370); iaIs32*	29.2±1.8	−10	45	0.9‡	3
*daf-2(e1370); iaIs34*	30.2±1.2	−7	65	0.9‡	3

* The *p* value was calculated by log-rank test as a comparison to *daf-16(mu86)* worms.

† The *p* value was calculated by log-rank test as a comparison to *skn-1(zu67)* worms.

‡ The *p* value was calculated by log-rank test as a comparison to *daf-2(e1370)* worms.

Longevity assays for *daf-2*-deficient strains were performed at 25°C. All other lifespan assays were conducted at 20°C.

SKN-1 is a transcription factor that mediates the phase II detoxification response during oxidative stress in *C. elegans* [27], and expression of constitutively active SKN-1 can extend lifespan via genetic pathways that are independent of DAF-16 [15]. We tested the hypothesis that a loss-of-function mutation in *skn-1* could abolish the prolongevity effects of HIF-1 over-expression. As shown in Fig. 3B and Table 2, over-expression of HIF-1 through *hif-1(P621G)* transgenes extended the lifespan of *skn-1(zu67)* mutants by up to 25%.

We then asked whether the longevity effect of increased HIF-1 protein was independent of the DAF-2 insulin-like signaling pathway by crossing the *hif-1(P621G)* transgene to a long-lived *daf-2* loss-of-function mutant. The *hif-1 (P621G)* transgene did not extend the lifespan of *daf-2(e1370)* worms (Fig. 3C, Table 2). Taken together, the genetic data indicate that increased dosage of HIF-1 promote longevity via a pathway that acts in parallel to DAF-16 and SKN-1, but may be downstream of the DAF-2 insulin-like receptor.

**Figure 4:**
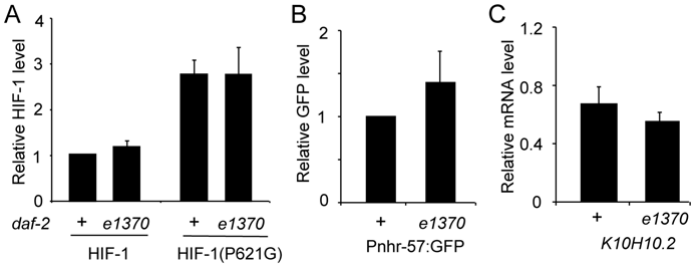
The *daf-2* insulin-like receptor does not regulate expression of HIF-1 protein or its targets. (A) A reduction of function mutation in *daf-2* does not change the protein levels of epitope-tagged HIF-1 (*iaIs28*) or HIF-1(P621G) (*iaIs32*) in young adult animals (*p*>0.2 from three independent experiments). (B,C) The expression of two HIF-1 targets were assayed in wild-type animals and in animals carrying a loss-of-function mutation in *daf-2*. (B) *Pnhr-57*:GFP expression was assayed by protein blots and was not significantly different in these genetic backgrounds (*p*>0.2 from three independent experiments) (B) Quantitative RT-PCR revealed a marginal differences in the levels of K10H10.2 mRNA (*p* = 0.09).

Studies in mammalian cell lines have shown that the insulin receptor pathway can influence translation of HIF-1α, and recent studies in *C. elegans* have suggested that DAF-16 might regulate HIF-1 expression in some developmental or environmental contexts [28], [29]. To directly test the hypothesis that DAF-2 regulated total HIF-1 protein levels, we compared the expression of epitope-tagged HIF-1 in wild-type animals and in *daf-2 (e1370)* mutants. We also assayed the expression of two HIF-1 targets: *Pnhr-57*:GFP and K10H10.2. Prior studies had shown that K10H10.2 mRNA levels were induced by hypoxia in wild-type animals in a *hif-1*-dependent manner and that mutations that caused over-expression of HIF-1 protein resulted in over-expression of K10H10.2 mRNA [2], [20]. As shown in Fig. 4A, the *daf-2 (e1370)* mutation did not have a significant effect on the expression levels of HIF-1 or HIF-1(P621G) proteins (Fig. 4A) or on the expression of two HIF-1 targets that we assayed (Figs. 4B, 4C).

### Effects of *hif-1* loss-of-function mutations on longevity

**Figure 5:**
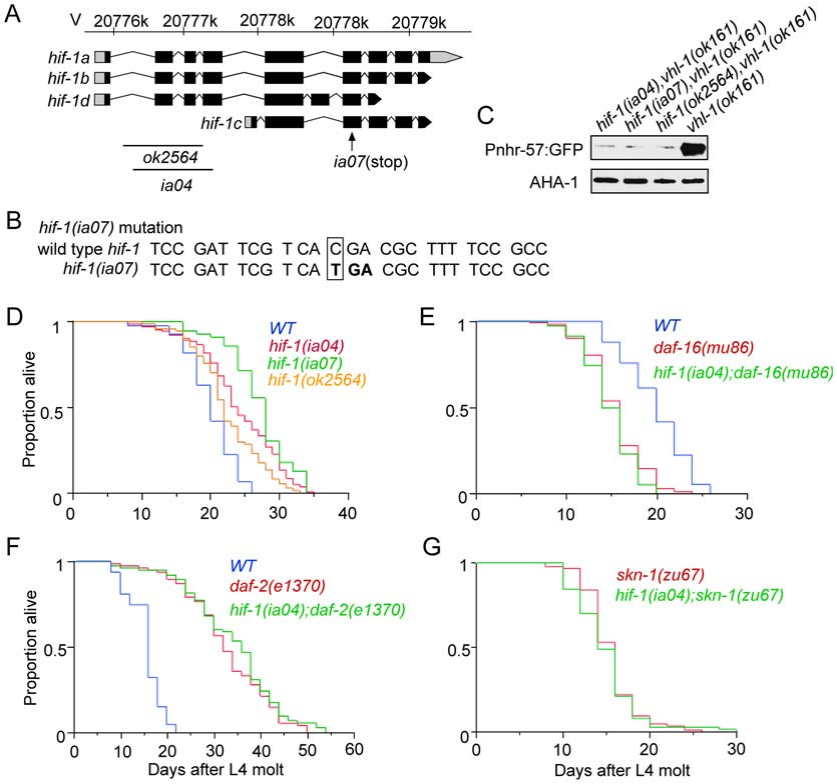
*hif-1* loss-of-function mutations extend the lifespan of wild-type animals, via pathway(s) that require *daf-16* and *skn-1*. (A,B) Characterization of *hif-1* mutation alleles. (A) The *hif-1* locus encodes 4 mRNA isoforms (www.wormbase.org). Exons are depicted as boxes, with coding sequences in black and untranslated regions in grey. The positions of two deletion alleles (*ia04* and *ok2564*) and one nonsense mutation (*ia07*) are shown. (B) The *hif-1 (ia07)* point mutation introduces an early stop codon; (C) Protein blots show that all three mutations in *hif-1* repress expression of the *Pnhr-57*::GFP reporter [20]. Expression was assayed in *vhl-1 (ok161)* mutants, which are defective in oxygen-dependent degradation of HIF-1. (D) *C. elegans* carrying *hif-1* loss-of-function mutations live longer than wild-type N2 (*p*<0.0001). (E) *hif-1(ia04);daf-16(mu86)* double mutant animals do not live longer than *daf-16(mu86)* single mutants. (F) *daf-2 (e1370)* single mutants and *daf-2 (e1370); hif-1(ia04)* double mutants have equivalent lifespans. (G) *hif-1(ia04);skn-1(zu67)* double mutant animals do not live longer than *skn-1(zu67)* single mutants.

Prior studies did not clearly predict the effects a *hif-1* loss-of-function mutation might have on longevity. HIF-1 had been shown to promote the expression of genes that inhibit formation of stress resistant dauer larvae. In accordance with this, *hif-1 (ia04)* mutants arrest larval development as partial dauers when cultured at 27°C [2]. Since mutations that confer stress resistance and dauer formation are often correlated with longer lifespan, it was reasonable to expect that *hif-1* mutants might age slowly. However, loss-of-function mutations in the *daf-16* or *skn-1* prolongevity genes are associated with premature aging [24], [26], [27], and our data in Fig. 2 had established that over-expression of HIF-1 extended lifespan. To address this question directly, we assayed the lifespans of wild-type animals and *hif-1 (ia04)* mutants. Under normal culture conditions (20°C), *hif-1 (ia04)* mutants lived approximately 20% longer than wild-type animals (24 days, compared to 20 days for wild-type N2) (Table 1). To further validate this finding, we isolated an additional loss-of-function allele in *hif-1* [*hif-1 (ia07)*, described in Figs. 5A and 5B]. Sequence analysis predicts that *hif-1 (ia07)* should encode a truncated protein lacking a transcriptional activation domain. We also characterized *hif-1 (ok2564)*, a deletion allele isolated by the *C. elegans* Genome Consortium (Figs. 5A). Phenotypic analyses confirmed that both *hif-1 (ia07)* and *hif-1 (ok2564)* decreased expression of HIF-1 targets (Fig. 5C). Each of the *hif-1* loss-of-function alleles increased the mean adult lifespan by at least 10% (Fig. 5D, Table 1). *hif-1* RNAi also extended the lifespan of wild-type animals (Table S2).

To understand whether the longevity phenotypes conferred by HIF-1 over-expression and *hif-1* loss-of-function mutations had similar genetic underpinnings, we asked whether the *hif-1(ia04)* mutation could extend the lifespan of *daf-16-* or *skn-1-*deficient mutants. As illustrated in Fig. 5E and 5F, *daf-16 (mu86)* animals and *daf-16 (mu86) hif-1 (ia04)* double mutants had similarly short lifespans (∼15 days), and there was not a significant difference between *daf-2(e1370)* and *daf-2(e1370) hif-1(ia04)* animals. We also determined that the *hif-1 (ia04)* deletion mutation did not extend the lifespan of *skn-1(zu67)* animals (Fig. 5G, Table 2). These data suggest that *hif-1* loss-of-function mutations extend life span via a mechanism that requires *daf-16* and *skn-1* function. Thus, the longevity phenotypes of HIF-1 over-expressing animals and *hif-1 (ia04)* animals have differing requirements for *daf-16* and *skn-1* function.

### Resistance to t-butyl-peroxide and heat stress

**Figure 6:**
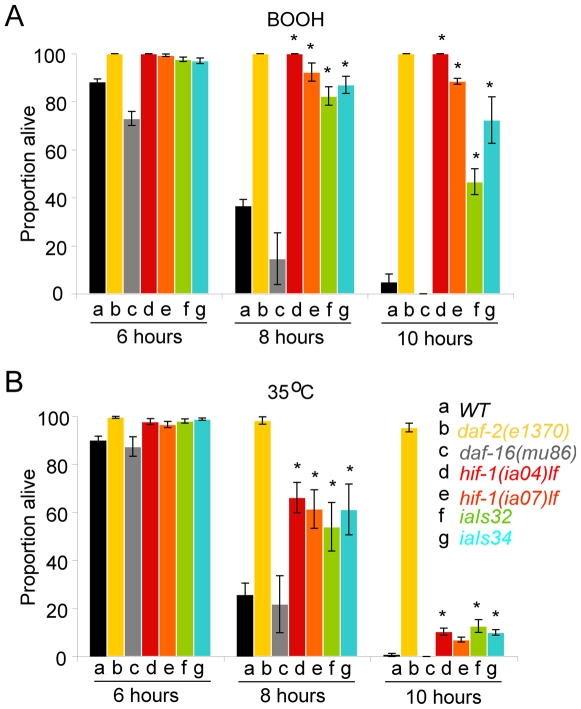
*hif-1* loss-of-function mutations and HIF-1 over-expression increase oxidative stress resistance and thermotolerance. (A, B) Resistance to (A) t-butyl peroxide or (B) heat stress was assayed in *hif-1* loss-of-function mutants and in animals over-expressing HIF-1. The *daf-2 (e1370)* and *daf-16 (mu86)* strains were included as a basis for comparison. Error bars represent the standard error of the means from at least 3 experiments. (*, *p*<0.0001 compared to wild type N2 worms using z-test).

Thermotolerance and stress resistance have been shown to be correlated with *C. elegans* longevity [30], [31]. To determine whether changes in *hif-1* dosage that extend lifespan also conferred resistance to oxidative stress, we assessed relative resistance to t-butyl-peroxide. We assayed two loss-of-function alleles of *hif-1* and two lines that expressed the stabilized HIF-1 (P621G). All of these strains exhibited significantly higher rates of survival in the oxidizing agent compared to wild-type (Fig. 6A). We also tested the ability of these strains to withstand heat stress. Prior studies had determined that both *hif-1 (ia04)* mutants and *egl-9-*deficient mutants were more resistant to heat stress than were wild-type animals [32]. We extended these studies and determined that, similar to the findings in peroxide treatment, loss-of-function mutations in *hif-1* or over-expression of HIF-1 increased the ability of the animals to survive at 35°C (Fig. 6B).

## Discussion

This study advances our understanding of the regulatory networks that link oxygen homeostasis and aging. Cells respond to oxygen deprivation by changing their patterns of gene expression to optimize energy production and mitigate cellular damage. Oxygen deprivation can extend the replicative lifespan of cells in culture [5], and *C. elegans* cultured in hypoxic conditions live longer [33]. The hypoxia-inducible factors have been termed master regulators of oxygen homeostasis [34], and in *C. elegans*, HIF-1 is required for most hypoxia-induced changes in gene expression [2]. HIF-1 targets include genes involved in metabolism, cell signaling, transcription and translation. We show here that over-expression of HIF-1 extends *C. elegans* lifespan in a dose-dependent manner. We hypothesize that multiple HIF-1 targets act in concert to slow cellular deterioration over time.

### Effects of HIF-1 over-expression

When over-expressed, HIF-1 acts in parallel to the stress responsive transcription factors DAF-16/FOXO and SKN-1/NRF to promote longevity. In wild-type animals, DAF-16 and SKN-1 are inhibited by the sole *C. elegans* insulin-like growth factor receptor, DAF-2. Analogous to mammalian IGF signaling pathways, DAF-2 regulates phosphoinositide 3-kinase (PI3K) signaling. The effectors of PI3K include kinases (AKT-1, AKT-2, and SGK-1) that phosphorylate DAF-16 and SKN-1 to repress transcription factor nuclear localization [15], [35], [36]. As illustrated in Fig. 3, HIF-1 over-expression can extend the lifespan of *daf-16* or *skn-1* loss-of-function mutants. Microarray studies have suggested that HIF-1 and DAF-16 may co-regulate some genes, but HIF-1 and DAF-16 clearly have independent functions as well [2], [37]. The relationship between HIF-1 and DAF-2 is less clear. The genetic data leave open the possibility that the prolongevity functions of HIF-1 are regulated by DAF-2. DAF-2 and the PI3K pathway have been shown to have other DAF-16-independent functions, including regulation of SKN-1 nuclear localization [15]. Additionally, mutations in *daf-2* were shown to protect *C. elegans* from the lethal effects of high-temperature anoxia [38], [39] and to confer resistance to hypoxia-induced defects in neural development [29]. Here, we have demonstrated that mutations in *daf-2* did not cause significant changes in whole animal levels of HIF-1 protein or in the expression of two HIF-1 targets that we assayed (Fig. 4), but DAF-2 may have tissue-specific or stage-specific roles in HIF-1 regulation.

A recent independent study from the Kaeberlein research group provides added support for the conclusion that HIF-1 over-expression extends *C. elegans* lifespan. Mehta et al reported that *vhl-1* or *egl-9* RNAi increased *C. elegans* longevity and decreased the toxic effects of transgenes that expressed polyglutamine repeats or beta amyloid. Further, they showed that *daf-16* RNAi did not completely suppress the longevity conferred by a *vhl-1* deletion mutation, and they found that *vhl-1* RNAi had no effect on the nuclear localization of DAF-16:GFP [40]. This further substantiates models in which HIF-1 over-expression acts in parallel to DAF-16 to modulate aging.

While moderate increases in HIF-1 protein expression promote longevity, dramatic over-expression of HIF-1 target genes can be detrimental. *C. elegans egl-9* mutants express HIF-1 targets at extremely high levels, and this causes a range of morphological and behavioral defects [19], [20], [41]. We and others have shown that animals carrying a strong loss-of-function mutation in *egl-9* do not live longer than wild-type animals (Table 1 and [42]). This suggests that animals treated with *egl-9* RNAi or animals carrying transgenes expressing stabilized HIF-1 benefit from increased HIF-1 function, but only to a point. Forcing gene expression beyond normal parameters can be deleterious. A similar pattern has been reported for yeast LAG1[43] and for *C. elegans* SKN-1. Moderate over-expression of SKN-1 extends life span, but over-expression from high copy transgenes is toxic [15].

### Effects of *hif-1* loss-of-function mutations

HIF-1 over-expression and *hif-1* loss-of-function mutations exert their prolongevity effects through different pathways. We demonstrated that three independently isolated *hif-1* mutations or depletion of *hif-1* by RNAi extended *C. elegans* lifespan. The *hif-1 (ia07)* allele is predicted to encode a truncated HIF-1 protein that lacks a transcriptional activation domain, and the *hif-1(ia07)* phenotype is slightly more severe than the phenotypes of the *hif-1 (ia04)* and *hif-1 (ok2564)* deletion alleles (Fig. 5A). In agreement with our findings, the Kapahi group determined that *hif-1* RNAi increased *C. elegans* lifespan, and they demonstrated that this effect was abrogated by dietary restriction [42]. These data show that the *hif-1* loss-of-function phenotype can be strongly influenced by nutrients.

There are also some intriguing differences between our findings and those from the other two groups that have recently examined the roles of HIF-1 in *C. elegans* aging. While we report and Chen et al [42] have reported that *hif-1-*deficient animals live longer when food is abundant, Mehta et al [40] did not detect differences between the lifespans of *hif-1 (ia04)* and wild-type animals in their experiments. This appears to reflect differences in assay conditions. Small differences in the bacterial food source, the temperature, or other conditions may alter the longevity phenotypes of *hif-1-*deficient animals. Chen et al conducted their experiments at 25°C, and they assayed the effects of varying amounts of live bacterial food on plates containing FUdR and antibiotics [42]. Mehta et al performed their experiments at 20°C and fed the animals UV-treated bacteria on plates containing FUdR and antibiotics [40]. All of the longevity assays that we report here were conducted in nutrient rich conditions in the absence of DNA synthesis inhibitors. Although our findings regarding the *hif-1* loss-of-function phenotype largely agree with those of Chen et al, there are open questions about the role of *daf-16* in *hif-1-*deficient animals. Chen et al found that *hif-1* RNAi could extend the mean lifespan of *daf-16 (mgDf47)* animals by 18%. In our experiments, double mutants carrying loss-of-function mutations in both *hif-1* and *daf-16* had similar lifespans to *daf-16 (mu86)* single mutants (Fig. 3).

### HIF-1 and hormesis

Why might apparently small differences in assay conditions influence the phenotypes of *hif-1-*deficient animals? The answer to this question may require a fuller understanding of the roles of HIF-1 in heat acclimation and hormesis. Prior studies demonstrated that wild-type *C. elegans* that have been incubated at 25°C were more thermotolerant than animals that had been incubated at 20°C. This process of heat acclimation required *hif-1* function [32]. This suggests that while loss-of-function mutations in *hif-1* or HIF-1 over-expression can increase thermal resistance (Fig. 6 and [32]), *hif-1-*deficient animals are less able to adapt to or benefit from changes in the environment. Future studies will investigate the ways in which culture conditions and environmental stresses can influence HIF-1 function and *hif-1-*deficient phenotypes and may discover additional roles for HIF-1 in aging, stress, and hormesis.

### Selective pressures to control HIF-1 signaling

To our knowledge, this is the first case in a multicellular organism in which wild-type animals (grown in nutrient-rich conditions) have been shown to live shorter than either loss-of-function mutants or animals over-expressing the protein. However, in yeast, both loss-of-function mutations in the LAG1 longevity assurance gene and moderate over-expression of LAG1 can extend replicative lifespan [43]. Other studies in mammalian cells have shown that similar phenotypes can be caused by loss-of-function or gain-of-function mutations that force a signaling pathway outside of its normal range. For example, in models for TNF-induced apoptosis, kinase-dead and constitutively active MK2 mutants have been shown to have similar effects on apoptosis [44]. When considering the complex roles of HIF-1 in aging, it is important to recognize that HIF-1 over-expression and *hif-1* loss-of-function phenotypes are qualitatively different and have distinct genetic requirements.

In the hundreds of millions of years since nematodes and mammals diverged from a common ancestor, some of the key molecules and feedback loops that modulated HIF signaling have been conserved [17], [45]. The EGL-9/VHL-1 pathway enables rapid up-regulation of HIF in hypoxic conditions, but the system is tightly controlled. In humans and in *C. elegans*, activation of HIF results in increased expression of the HIF prolyl hydroxylases (PHD/EGL-9) [2], [18]. This establishes negative feedback loops to attenuate HIF expression. While either *hif-1* depletion or HIF-1 over-expression increase longevity in optimal lab culture conditions, the conservation of HIF regulatory networks suggests that there is a strong selective pressure for keeping HIF signaling within a normal range, where HIF can effectively respond to subtle changes in the environment.

Small molecule inhibitors of the HIF prolyl hydroxylases are being developed as treatments for anemia or other pathologies that may be ameliorated by increased HIF expression. There is also great interest in pharmaceuticals that can inhibit HIF and may be effective in combinatorial treatments for certain cancers [46]. We anticipate that drugs that target HIF may have widespread, dose-dependent impacts on stress resistance and on somatic aging.

## Materials and Methods

### *C. elegans* strains

The mutant alleles were as follows: LGI: *daf-16(mu86)lf*; LGII: *daf-2(e1370) reduction-of-function*; LGV: *hif-1(ia04)lf, hif-1(ia07)lf, hif-1(ok2564)lf*, *egl-9(sa307)lf*; LGX: *vhl-1(ok161)lf*.

### *hif-1* minigenes and generation of transgenic animals

The pFOX1 and pFOX4 constructs encode wild-type HIF-1 and HIF-1(P621G), respectively. pFOX1 includes 5.2 kb genomic sequence 5′ to the *hif-1* coding region. The first exon and first intron of *hif-1* are fused to a cDNA fragment including exon 2 to exon 9 of *hif-1* (for the predominant mRNA isoform: *hif-1a*). Five copies of c-myc epitope (5×myc) (from clone CD3-128, Arabidopsis Biology Resources Center) were inserted C-terminal to *hif-1* coding sequences, and this was followed by a stop codon and 400 bp of genomic sequence just 3′ to the *hif-1* coding region. To create pFOX4, the P621G point mutation was introduced to the pFOX1 sequence. Microparticle bombardment was used to create *C. elegans* carrying integrated copies of each construct. The *hif-1* constructs were introduced to *hif-1 (ia04), unc-119(ed3)* animals, and the *unc-119* rescuing plasmid pPD#MM016b was the co-transformation marker [47]. Animals carrying integrated copies of the transgenes were isolated and backcrossed at least four times prior to further characterization.

### Lifespan and stress analyses

Longevity assays were conducted as described previously [24] unless noted otherwise. Prior to life span assays, all strains were maintained at 20°C for at least two generations with abundant food. The OP50 bacterial food was grown in L-broth to a 600 nm optical density of approximately 0.4, and 250 microliters of live bacterial culture was spotted onto each 60 mm NGM plate. The plates did not contain FUdR or antibiotics. One to three days later, young adult hermaphrodites were allowed to lay eggs overnight on the bacterial food. The L4 larvae grown from these eggs were transferred to fresh plates. Each plate included 20–25 animals, and at least 50 animals were used in each independent trial. Longevity assays for *daf-2-*deficient strains were performed at 25°C. All other lifespan assays were conducted at 20°C. The worms were transferred to new plates every two days for the first two weeks and every six days thereafter. The data from individual experiments are listed in Table S1. Any experiments in which the assay conditions were modified are explicitly noted in the text and in the legends for Tables S2 and S3. Viability was scored every 2 days. Worms that crawled off the plates, burst at the vulva or died because progeny hatched in utero were excluded from final statistical analyses. Life span statistical analyses were carried out using JMP software (version 7.0) to determine the means and percentiles. *p* values were calculated using the log-rank (Mantel-Cox) method.

In oxidative stress assays, young adult worms (one day after L4 molt) were transferred to NGM plates containing 7.2 mM t-butyl-peroxide (Sigma) and OP50 food. Animals were incubated at 20°C and were scored for survival at the time points shown. For thermal stress assays, young adult worms were incubated at 35°C on NGM plates seeded with OP50 and were scored for survival. At least 50 worms were tested in each trial and at least three independent trials were performed for each stress assay.

### Protein blots and quantitative RT-PCR

For each lane of an immunoblot, ten (for *Pnhr57*:GFP) to 100 (for the epitope-tagged HIF-1) L4 or young adult worms were assayed. Mouse anti-AHA-1 antibody (cell culture supernatant) [8] was produced in ISU Hybridoma Facility and used at 1∶100. Mouse anti-myc ascites (clone 9E10) from the Developmental Studies Hybridoma Bank was used at 1∶1000. Mouse anti-GFP antibody (from Roche) was used at 1∶1000.

Quantitative RT-PCR was performed as described previously [20]. *inf-1* is not regulated by hypoxia and was used as an input control. The standard curve method was used to analyze the expression levels. Student t-tests were used to determine the *p* value for protein expression and quantitative RT-PCR data.

## Supporting Information

Table S1 Data from individual lifespan experiments
(0.16 MB DOC)

Table S2 Effects of hif-1 RNAi on the longevity of wildtype worms
(0.06 MB DOC)

Table S3 Lifespan assays at 25°C on UV-irradiated bacterial food (OP50)
(0.04 MB DOC)
