# Supplementary material for: The HIF-1 Hypoxia-Inducible Factor Modulates Lifespan in C. elegans
Source: PLoS One. 2009 Jul 27;4(7):e6348. doi: 10.1371/journal.pone.0006348 (PMC2711329; doi:10.1371/journal.pone.0006348)
Supplement: Table S1 — Data from individual lifespan experiments (0.16 MB DOC) [file pone.0006348.s001.doc]

**Table S1: Data from individual lifespan experiments**

| **Genotype** | **life span**  **mean +S.E.** | **Percentage *v*. control** | **Maximum lifespan** | **n** | ***p* value*** |
| --- | --- | --- | --- | --- | --- |
| N2 wild-type | 19.9 ± 0.5 |  | 26 | 41 |  |
| N2 wild-type | 20.3 ± 0.6 |  | 26 | 39 |  |
| N2 wild-type | 20.0 ± 0.6 |  | 26 | 41 |  |
| N2 wild-type | 20.4 ± 0.5 |  | 30 | 45 |  |
| *iaIs27* [*Phif-1::hif-1::myc]* | 23.5 ± 0.5 | 18***** | 34 | 47 | <0.0001***** |
| *iaIs27* [*Phif-1::hif-1::myc]* | 21.3 ± 0.8 | 7***** | 36 | 45 | 0.04***** |
| *iaIs27* [*Phif-1::hif-1::myc]* | 21.4 ± 1.2 | 7***** | 36 | 17 | 0.03***** |
| *iaIs28 [Phif-1::hif-1::myc]* | 24.6 ± 0.6 | 23***** | 36 | 46 | <0.0001***** |
| *iaIs28 [Phif-1::hif-1::myc]* | 22.0 ± 0.6 | 10***** | 28 | 40 | 0.003***** |
| *iaIs28 [Phif-1::hif-1::myc]* | 22.2 ± 0.4 | 11***** | 30 | 45 | 0.001***** |
| *iaIs32 [Phif-1::hif-1(P621G)::myc]* | 27.2 ± 0.7 | 36***** | 36 | 41 | <0.0001***** |
| *iaIs32 [Phif-1::hif-1(P621G)::myc]* | 27.0 ± 1.1 | 35***** | 38 | 39 | <0.0001***** |
| *iaIs32 [Phif-1::hif-1(P621G)::myc]* | 23.4 ± 0.9 | 17***** | 36 | 37 | <0.0001***** |
| *iaIs33 [Phif-1::hif-1(P621G)::myc]* | 25.3 ± 0.8 | 27***** | 36 | 42 | <0.0001***** |
| *iaIs33 [Phif-1::hif-1(P621G)::myc]* | 24.7 ± 1.2 | 23***** | 40 | 41 | <0.0001***** |
| *iaIs33 [Phif-1::hif-1(P621G)::myc]* | 22.2 ± 0.7 | 11***** | 34 | 42 | 0.001***** |
| *iaIs34 [Phif-1::hif-1(P621G)::myc]* | 28.3 ± 0.6 | 42***** | 38 | 36 | <0.0001***** |
| *iaIs34 [Phif-1::hif-1(P621G)::myc]* | 27.3 ± 0.8 | 37***** | 38 | 43 | <0.0001***** |
| *iaIs34 [Phif-1::hif-1(P621G)::myc]* | 24.9 ± 0.5 | 25***** | 34 | 46 | <0.0001***** |
| *hif-1(ia04)* | 25.7 ± 1.1 | 34***** | 34 | 27 | <0.0001***** |
| *hif-1(ia04)* | 24.2 ± 0.7 | 21***** | 34 | 43 | <0.0001***** |
| *hif-1(ia04)* | 25.6 ± 0.8 | 28***** | 32 | 44 | <0.0001***** |
| *hif-1(ia04)* | 21.5 ± 0.7 | 8***** | 32 | 33 | 0.002***** |
| *hif-1(ia07)* | 26.6 ± 1.0 | 33***** | 36 | 29 | <0.0001***** |
| *hif-1(ia07)* | 27.2 ± 0.9 | 36***** | 38 | 33 | <0.0001***** |
| *hif-1(ok2564)* | 22.8 ± 0.6 | 14***** | 30 | 43 | <0.0001***** |
| *hif-1(ok2564)* | 22.2 ± 0.7 | 11***** | 31 | 31 | <0.0001***** |
| *egl-9(sa307)* | 21.8 ± 0.7 | 9***** | 29 | 32 | 0.007***** |
| *egl-9(sa307)* | 21.5 ± 0.5 | 8***** | 26 | 27 | 0.007***** |
| *egl-9(sa307)* | 20.5 ± 0.7 | 3***** | 30 | 34 | 0.45***** |
| *egl-9(sa307)* | 19.6 ± 0.6 | -2***** | 28 | 38 | 0.41***** |
| *egl-9(sa307);hif-1(ia04)* | 18.7 ± 0.5 | -7***** | 24 | 45 | 0.3***** |
| *egl-9(sa307);hif-1(ia04)* | 20.7 ± 0.4 | 4***** | 26 | 47 | 0.8***** |
| *egl-9(sa307);hif-1(ia04)* | 18.1 ± 0.4 | -9***** | 26 | 42 | 0.0003***** |
| *daf-16(mu86)* | 15.6 ± 0.6 |  | 22 | 41 |  |
| *daf-16(mu86)* | 15.4 ± 0.5 |  | 20 | 37 |  |
| *daf-16(mu86)* | 15.1 ± 0.6 |  | 24 | 34 |  |
| *daf-16(mu86)* | 15.2 ± 0.4 |  | 20 | 40 |  |
| *daf-16(mu86)* | 14.1 ± 0.4 |  | 20 | 39 |  |
| *daf-16(mu86); hif-1(ia04)* | 13.7 ± 0.4 | -12^†^ | 20 | 41 | 0.003^†^ |
| *daf-16(mu86); hif-1(ia04)* | 14.5 ± 0.4 | -6^†^ | 20 | 33 | 0.14^†^ |
| *daf-16(mu86); hif-1(ia04)* | 16.6 ± 0.4 | 10^†^ | 20 | 30 | 0.11^†^ |
| *daf-16(mu86); iaIs32* | 19.8 ± 0.6 | 30^†^ | 26 | 41 | <0.0001^†^ |
| *daf-16(mu86); iaIs32* | 18.1 ± 0.7 | 28^†^ | 26 | 36 | <0.0001^†^ |
| *daf-16(mu86); iaIs34* | 20.3 ± 0.6 | 33^†^ | 28 | 40 | <0.0001^†^ |
| *daf-16(mu86); iaIs34* | 20.2 ± 0.6 | 33^†^ | 26 | 38 | <0.0001^†^ |
| *skn-1(zu67)* | 15.1 ± 0.3 |  | 22 | 58 |  |
| *skn-1(zu67)* | 16.1 ± 0.7 |  | 26 | 29 |  |
| *skn-1(zu67);hif-1(ia04)* | 14.6 ± 0.4 | -3^‡^ | 20 | 52 | 0.57^‡^ |
| *skn-1(zu67);hif-1(ia04)* | 15.5 ± 1.1 | -4^‡^ | 30 | 24 | 0.8^‡^ |
| *skn-1(zu67); iaIs32* | 19.1 ± 0.5 | 26^‡^ | 28 | 58 | <0.0001^‡^ |
| *skn-1(zu67); iaIs32* | 19.4 ± 0.7 | 20^‡^ | 30 | 40 | <0.0001^‡^ |
| *skn-1(zu67); iaIs34* | 16.3 ± 0.4 | 8^‡^ | 26 | 49 | 0.002^‡^ |
| *skn-1(zu67); iaIs34* | 18.7 ± 0.7 | 16^‡^ | 30 | 59 | <0.0001^‡^ |
| *daf-2(e1370)* | 29.6 +2.0 |  | 50 | 23 |  |
| *daf-2(e1370)* | 32.6 +1.7 |  | 48 | 24 |  |
| *daf-2(e1370)* | 34.9 +1.7 |  | 50 | 29 |  |
| *daf-2(e1370); hif-1(ia04)* | 28.7 +3.1 | -3^¥^ | 54 | 15 | 0.77^¥^ |
| *daf-2(e1370); hif-1(ia04)* | 35.5 +1.6 | 9^¥^ | 54 | 30 | 0.18^¥^ |
| *daf-2(e1370); hif-1(ia04)* | 35.3 +1.7 | 5^¥^ | 54 | 30 | 0.9^¥^ |
| *daf-2(e1370); iaIs32* | 35.8 +3.2 | 16^¥^ | 58 | 17 | 0.007^¥^ |
| *daf-2(e1370); iaIs32* | 25.2 +1.7 | -16^¥^ | 40 | 28 | 0.6^¥^ |
| *daf-2(e1370); iaIs34* | 31.5 +1.2 | 3^¥^ | 54 | 21 | 0.6^¥^ |
| *daf-2(e1370); iaIs34* | 31.2 +2.0 | 2^¥^ | 52 | 23 | 0.6^¥^ |
| *daf-2(e1370); iaIs34* | 27.8 +2.4 | -10^¥^ | 42 | 21 | 0.42^¥^ |

* The longevity of was compared to wild-type N2, and the *p* values were calculated by log-rank tests.

† The longevity of was compared to *daf-16(mu86)* worms, and the *p* values were calculated by log-rank tests.

‡ The longevity of was compared to *skn-1(zu67)* worms, and the *p* values were calculated by log-rank tests.

¥ The longevity of was compared to *daf-2(e1370)* worms, and the *p* values were calculated by log-rank tests.

The experiments in Table S1 were conducted at 20^o^C on live bacterial food, as described in the Materials and Methods of the main text, with the exception of the strains including the *daf-2(e1370)* mutation, which were assayed at 25^o^C.
