# Supplementary material for: The HIF-1 Hypoxia-Inducible Factor Modulates Lifespan in C. elegans
Source: PLoS One. 2009 Jul 27;4(7):e6348. doi: 10.1371/journal.pone.0006348 (PMC2711329; doi:10.1371/journal.pone.0006348)
Supplement: Table S2 — Effects of hif-1 RNAi on the longevity of wildtype worms (0.06 MB DOC) [file pone.0006348.s002.doc]

**Table S2:** **Effects of *hif-1* RNAi on the longevity of wildtype worms.**

| **strain** | **Adult life span**  **mean +S.E.** | **Maximum lifespan** | **Percentage *v*. control RNAi** | **n** | ***p***^†^ **value** |
| --- | --- | --- | --- | --- | --- |
| *N2 (L4440 RNAi)* | 16.5 ± 0.7 | 28 |  | 45 |  |
| *N2 (L4440 RNAi)* | 17.3 ± 0.7 | 28 |  | 42 |  |
| *N2 (L4440 RNAi)* | 16.7 ± 0.5 | 26 |  | 66 |  |
| *N2 (hif-1 RNAi)* | 19.5 ± 0.8 | 30 | 15 | 35 | 0.009 |
| *N2 (hif-1 RNAi)* | 19.6 ± 0.9 | 32 | 16 | 43 | 0.0008 |
| *N2 (hif-1 RNAi)* | 19.0 ± 0.7 | 28 | 12 | 39 | 0.02 |

These lifespan assays were performed at 20ºC.

† The *p* value was calculated by log-rank test as a comparison to N2 worms treated with control RNAi (L4440).

**Supplemental Methods for Table S2.**

*hif-1* RNAi constuct and lifespan analyses on RNAi bacterial food: To generate the *hif-1* RNAi construct, a 1.4 kb *hif-1* cDNA fragment was excised from pHJ06 (Jiang et al. 2001 PNAS 98: 7916) and was inserted into the pPD129.36 (L4440) vector at the EcoRV and XhoI sites. The *hif-1* RNAi construct was transformed into HT115 bacterial strain. RNAi plates used in life span assay were prepared with 5mM IPTG and with no FuDR or antibiotics (Kamath et al 2001 Genome Biol 2: RESEARCH0002). The lifespan assays were performed at 20ºC. Young adult hermaphrodites which had been maintained at 20ºC on NGM plates and OP50 bacterial food were transferred to RNAi plates and allowed to lay eggs overnight. The L4 larvae grown from these eggs were transferred to fresh plates. Each plate included 20 – 25 animals, and at least 50 animals were used in each independent trial. The worms were transferred to new RNAi plates every two days for the first two weeks and every six days thereafter. Viability was scored every 2 days. Worms that crawled off the plates, burst at the vulva or died because progeny hatched in utero were excluded from final statistical analyses. Life span statistical analyses were carried out using JMP software (version 7.0) to determine the means and percentiles.
