# Supplementary material for: The HIF-1 Hypoxia-Inducible Factor Modulates Lifespan in C. elegans
Source: PLoS One. 2009 Jul 27;4(7):e6348. doi: 10.1371/journal.pone.0006348 (PMC2711329; doi:10.1371/journal.pone.0006348)
Supplement: Table S3 — Lifespan assays at 25°C on UV-irradiated bacterial food (OP50) (0.04 MB DOC) [file pone.0006348.s003.doc]

**Table S3. Lifespan assays at 25 ºC on UV-irradiated bacterial food (OP50).**

| **strain** | **Adult life span**  **mean +S.E.** | **Maximum lifespan** | **Percentage *v*. N2** | **n** | ***p**** **value** |
| --- | --- | --- | --- | --- | --- |
| *N2* | 15.9 ± 0.5 | 22 |  | 30 |  |
| *N2* | 15.3 ± 0.5 | 20 |  | 41 |  |
| *hif-1(ia04)* | 17.5 ± 0.4 | 22 | 13 | 33 | 0.001 |
| *hif-1(ia04)* | 17.3 ± 0.4 | 26 | 11 | 72 | 0.0003 |

* The *p* value was calculated by log-rank test as a comparison to N2 worms.

**Supplemental Methods for Table S3.**

To kill the bacterial food, standard NGM plates spotted with OP50 bacteria as described in the main text were exposed to UV light (302nm) for 15 minutes using a Benchtop UV transilluminator system (UVP). The death of UV irradiated bacterial food was confirmed by streaking the bacteria on LB agar plates. For the experiments in Table S3, young adult hermaphrodites which had been maintained at 20ºC on NGM plates spotted with live OP50 bacterial food were allowed to lay eggs overnight on the UV-irradiated bacterial food. The L4 larvae grown from these eggs at 20ºC were transferred to fresh UV-irradiated plates and removed to 25ºC to perform the lifespan assays as described in the main text.
